# Endothelial cells microparticle-associated protein disulfide isomerase promotes platelet activation in metabolic syndrome

**DOI:** 10.18632/oncotarget.13081

**Published:** 2016-11-04

**Authors:** Guan-qi Fan, Ran-ran Qin, Yi-hui Li, Dai-jun Song, Tong-shuai Chen, Wei Zhang, Ming Zhong, Yun Zhang, Yan-qiu Xing, Zhi-hao Wang

**Affiliations:** ^1^ The Key Laboratory of Cardiovascular Remodeling and Function Research, Chinese Ministry of Education and Chinese Ministry of Health, The State and Shandong Province Joint Key Laboratory of Translational Cardiovascular Medicine, Department of Cardiology Qilu Hospital of Shandong University, Ji'nan 250012, P.R. China; ^2^ Department of Radiology Medicine, Qilu Hospital of Shandong University, Ji'nan 250012, P.R. China; ^3^ Department of Geriatrics, Qilu Hospital of Shandong University, Ji'nan 250012, P.R.China; ^4^ Department of Emergency, Donggang People's Hospital, Rizhao, 276800, P.R. China

**Keywords:** protein disulfide isomerase, platelet activation, insulin resistance, endothelial microparticles

## Abstract

**Background:**

Metabolic syndrome (MetS) is a common challenge in the world, and the platelet activation is enhanced in MetS patients. However, the fundamental mechanism that underlies platelet activation in MetS remains incompletely understood. Endothelial cells are damaged seriously in MetS patients, then they release more endothelial microparticles (EMPs). After all, whether the EMPs participate in platelet activation is still obscure. If they were, how did they work?

**Results:**

We demonstrated that the levels of EMPs, PMPs (platelet derived microparticles) and microparticle-carried-PDI activity increased in MetS patients. IR endothelial cells released more EMPs, the EMP-PDI was more activated. EMPs can enhance the activation of CD62P, GPIIb/IIIa and platelet aggregation and this process can be partly inhibited by PDI inhibitor such as RL90 and rutin. Activated platelets stimulated by EMPs expressed more PDI on cytoplasm and released more PMPs.

**Materials and Methods:**

We obtained plasma from 23 MetS patients and 8 normal healthy controls. First we built insulin resistance (IR) model of human umbilical vein endothelial cells (HUVECs), and then we separated EMPs from HUVECs culture medium and used these EMPs to stimulate platelets. Levels of microparticles, P-selectin(CD62P), Glycoprotein IIb/IIIa (GPIIb/IIIa) were detected by flow cytometry and levels of EMPs were detected by enzyme-linked immunosorbent assay (ELISA). The protein disulfide isomerase (PDI) activity was detected by insulin transhydrogenase assay. Platelet aggregation was assessed by turbidimetry.

**Conclusion:**

EMPs can promote the activation of GPIIb/IIIa in platelets and platelet aggregation by the PDI which is carried on the surface of EMPs.

## INTRODUCTION

MetS caused by IR induces hyperinsulinemia and results in a group of diseases that can cause the atherosclerosis [1]. MetS promotes platelet activation. MetS accelerates the risk of type 2 diabetes (T2DM) and cardiovascular disease considerably and it is a major cause of thrombosis, which can potentially lead to cardio-cerebrovascular diseases and is related to cancer [2–4]. Thrombosis is closely related to organizational factors, platelet activation and fibrin formation, in which platelet activation is the key step of thrombosis. Platelet activation is a process of signaling cascade. The mechanism of how platelets are activated in the early process in MetS is unclear.

PDI is a member of the thioredoxin superfamily of redoxproteins. PDI has three catalytic activities including thiol-disulfide oxidoreductase, disulfide isomerization and redox-dependent chaperone [5]. There are studies that suggest PDI may participate in platelet early activation [6]. It has been established that PDI is secreted from a variety of cell types, recently extracellular PDI has been found in platelet surface, endothelial cells and leukocytes [7]. The a and a ' domains of PDI are homologous to thioredoxin and each domain contains an independent active site. Each active site contains two cysteines in the sequence WCGHCK which mediates PDI's activities [8]. However, how PDI participates in the platelet early activation in MetS has not been reported.

There are multiple ways of platelet activation. GPIIb/IIIa (also named integrin α_IIb_β_3_) mediates the final way of platelet activation [9, 10]. GPIIb/IIIa receptor activation depends on the disulfide bond reduction and β3 integrin space-conformation change [11]. Furthermore, PDI can catalysis disulfide bond reduction of GPIIb/IIIa receptor and increase the number of sulfhydryl-free, thereby activating GPIIb/IIIa receptor into high affinity state, improving the ability to combine GPIIb/IIIa receptor with fibrinogen [12, 13]. It is unclear whether platelets can be activated by PDI through the above way and where the PDI is from in MetS.

Membrane microparticles (MP) are shed by endothelial and blood cells upon activation or apoptosis, under the form of membrane vesicles ranging in size from 0.1 to 1 μm, and mostly expressing phosphatidylserine. Endothelial microparticles (EMPs) are vesicles generated by exocytic budding and display surface antigens from endothelial cells [14]. Some studies have shown that endothelial cells surface carry PDI, which can activate β1 and β3 integrin on platelets [15]. Platelet surface PDI (psPDI) has been studied extensively for its role in platelet activation and aggregation [12, 16]. Platelet microparticle-associated PDI plays an important role in the platelet aggregation in T2DM and can worsen IR [17]. Therefore, we hypothesis that the endothelial cells can release PDI-containing EMPs, the PDI can activate GPIIb/IIIa and GPIIb/IIIa is involved in platelet early activation. Meanwhile platelets released the PMPs. These steps can result in the progress of platelet signaling cascade in the condition of MetS.

## RESULTS

### Comparison of microparticles and PDI activity in plasma

**Figure 1:**
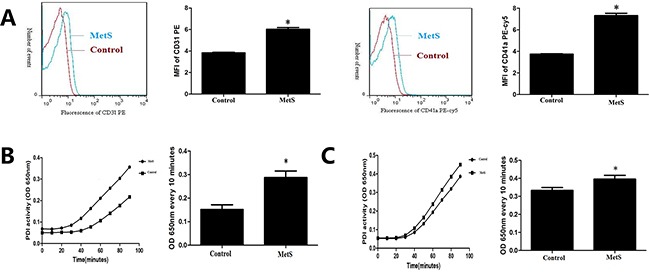
Comparison of microparticle and PDI activity in plasma **A.** Detection of endothelial microparticles (EMPs) and platelet derived microparticles (PMPs) by flow cytometry in plasma of the control group and MetS patients. **B.** Detection of plasma PDI activity by insulin transhydrogenase assay of the control group and MetS patients. **C.** Detection of PDI activity in plasma microparticles of the control group and MetS patients. *P<0.05 compared with the control. Data are from at least three separate experiments.

As shown in Figure 1, the expression of CD31 (6.03±0.27 vs. 3.84±0.09, *P*< 0.05) and CD41a (7.32±0.37 vs. 3.74±0.08, *P*< 0.05) in plasma microparticles of MetS patients were significantly higher than the control (Figure 1A), which suggested that EMPs and PMPs increased in MetS blood circulation.

Compared with normal control, PDI enzymatic reaction curve of MetS plasma had steeper slope, rised faster, and changes of OD650nm every 10 minutes were higher (*P*<0.05) (Figure 1B). PDI enzymatic reaction curve of MetS plasma microparticles had steeper slope, rised faster, and changes of OD650nm every 10 minutes were higher than normal control (*P*< 0.05) (Figure 1C).

### Build IR HUVECs model

**Figure 2:**
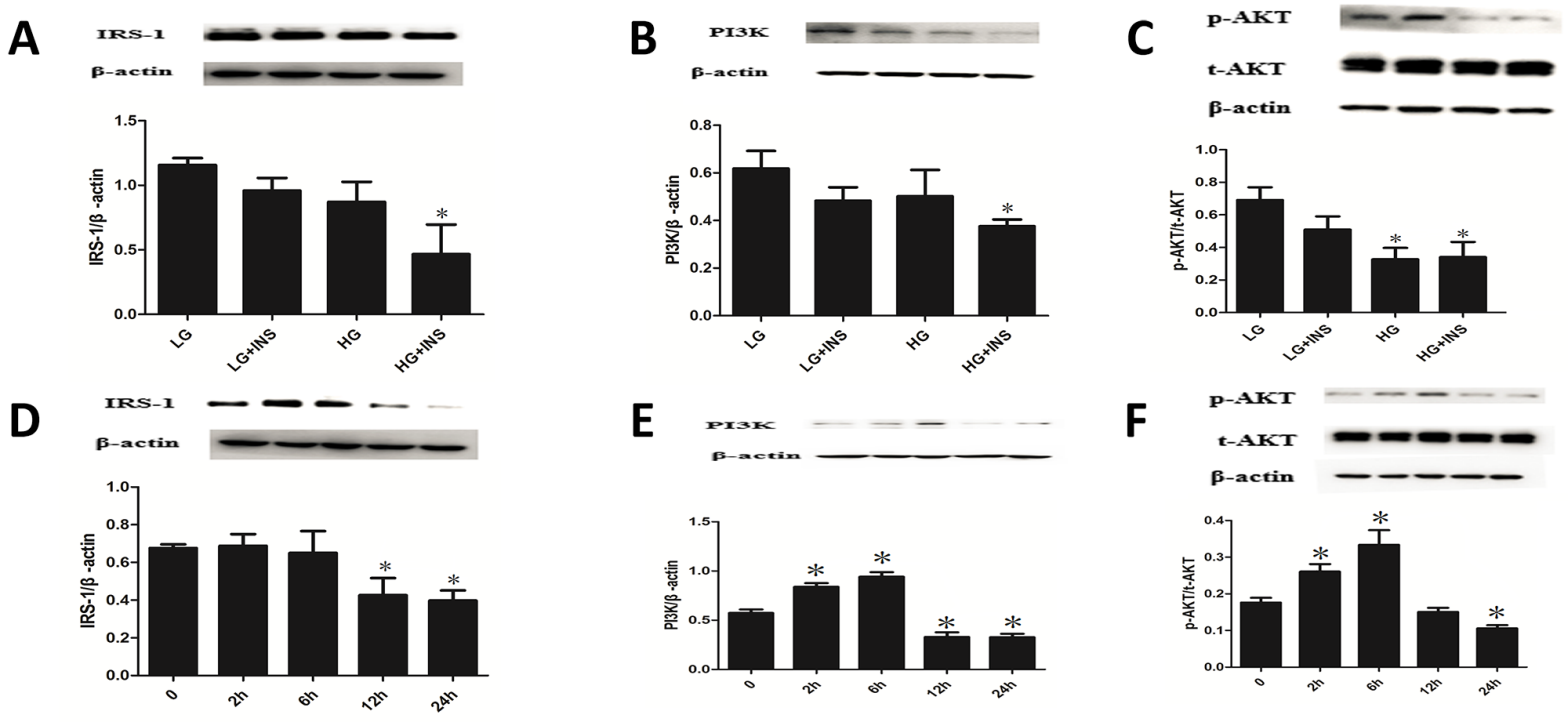
Build HUVECs IR model **A-C.** Culture HUVECs by low glucose DMEM complete medium under conditions of low glucose(LG), low glucose combined with high insulin(LG+INS), high glucose(HG), high glucose combined with high insulin (HG+INS) for 24 hours. The expression of IRS1, PI3K and p-Akt were detected by Western blotting analysis. *P < 0.05 compared with control. **D-F.** Culture HUVECs under high glucose combined with high insulin for 0, 2h, 6h, 12h, 24h,the expression of IRS1,PI3K and p-Akt were detected by Western blotting analysis. *P < 0.05 compared with 0h. Data are means ± SD from at least three separate experiments.

Culture HUVECs under conditions of low glucose, low glucose combined with high insulin, high glucose and high glucose combined with high insulin. HUVECs displayed a cobblestone-like shape. There were no morphological differences between all condition HUVECs (Supplementary Figure S1). Compared with low glucose group, low glucose combined with high insulin group had similar expression of IRS1, PI3K and p-Akt (*P*>0.05) (Figure 2A–2C). Compared with low glucose group, high glucose group had similar expression of IRS1 and PI3K (*P*>0.05), but lower expression of p-Akt *(P*<0.05) (Figure 2A–2C). Compared with low glucose group, high glucose combined with high insulin group had lower expression of IRS1, PI3K and p-Akt (*P*<0.05) (Figure 2A–2C). Therefore, we considered high glucose combined with high insulin as condition of building IR model.

Culture HUVECs under condition of high glucose combined with high insulin for 0, 2h, 6h, 12h, 24h. Expression of IRS1 didn't change at 2h and 6h, decreased significantly from 12h (*P*<0.05) and lasted stable for 24h (Figure 2D). PI3K and p-Akt, downstream of IRS1, changed in a trend of increase at first and decrease after. The inflection point happened at 6h (Figures 2E–2F). These results suggested that IR happened at 12h when culturing HUVECs under high glucose combined with high insulin condition and still existed at 24h. According to growth state of HUVECs, we considered high glucose combined with high insulin and 24h as condition of building IR HUVECs model.

### IR HUVECs released EMPs carrying PDI

**Figure 3:**
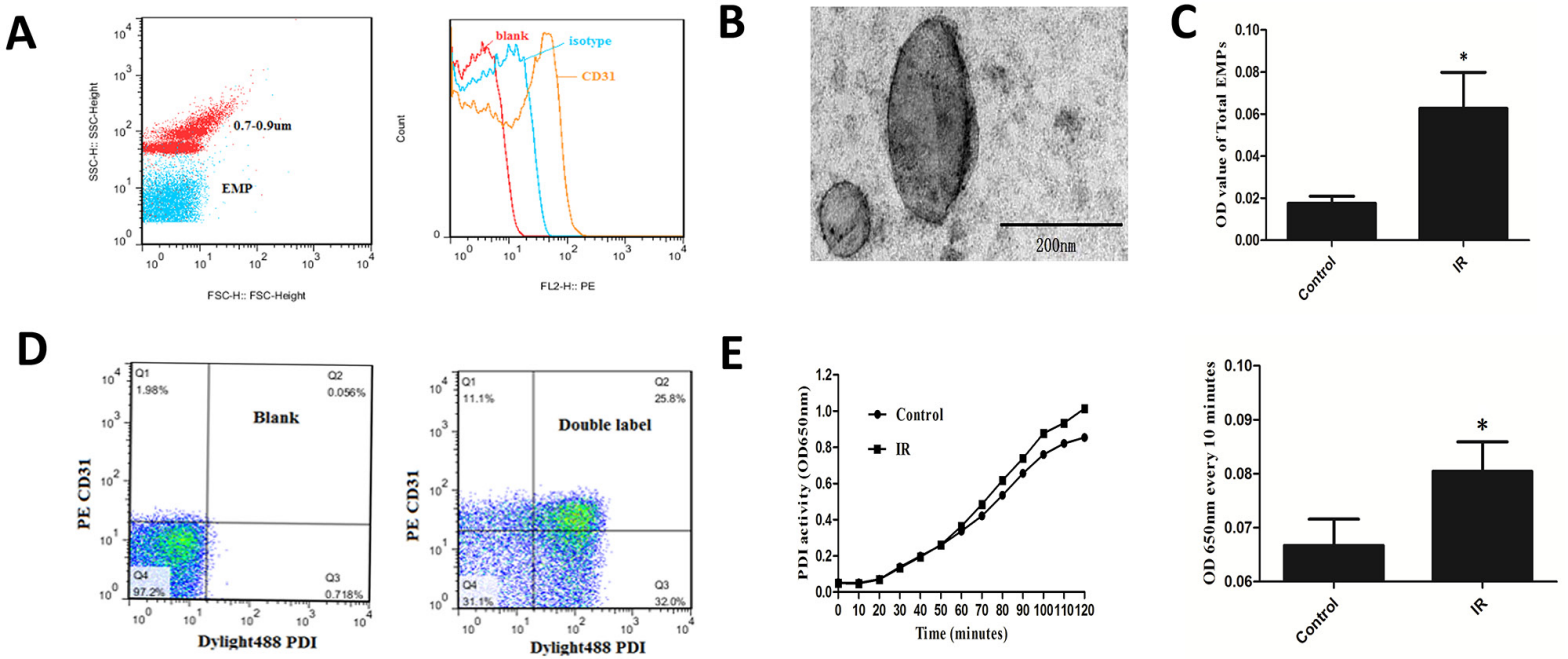
Detection of EMPs quantity and EMP-PDI activity **A.** Define gate of microparticles using standard fluorescent microspheres with 0.7-0.9μm. CD31 represented endothelial cell derivation. **B.** TEM images of EMPs. Scale bars 200nm. **C.** ELISA detected the number of EMPs in supernatant of IR and normal HUVEC medium.**D.** Bicolor flow cytometry confirmed EMP carrying PDI. **E.** ELISA combined with insulin transhydrogenase assay detected PDI activity of EMPs. *P < 0.05 compared with control. Data are means ± SD from at least three separate experiments.

Define gate of microparticles using standard fluorescent microspheres with 0.7-0.9μm CD31 represented endothelial cell derivation (Figure 3A). Transmission electron microscopy (TEM) images demonstrated intact EMPs of various sizes (Figure 3B). IR HUVEC released more EMPs (*P*<0.05) by approximately 3 times than that of normal control (Figure 3C). Bicolor flow cytometry confirmed that PDI was carried by EMPs (Figure 3D). PDI enzymatic reaction curve of EMPs in supernatant of IR HUVEC medium had steeper slope, rised faster, and changes of OD650nm every 10 minutes were higher than control (*P*< 0.05) (Figure 3E). This suggested that IR EMPs had higher PDI activity.

### EMPs activated platelet

**Figure 4:**
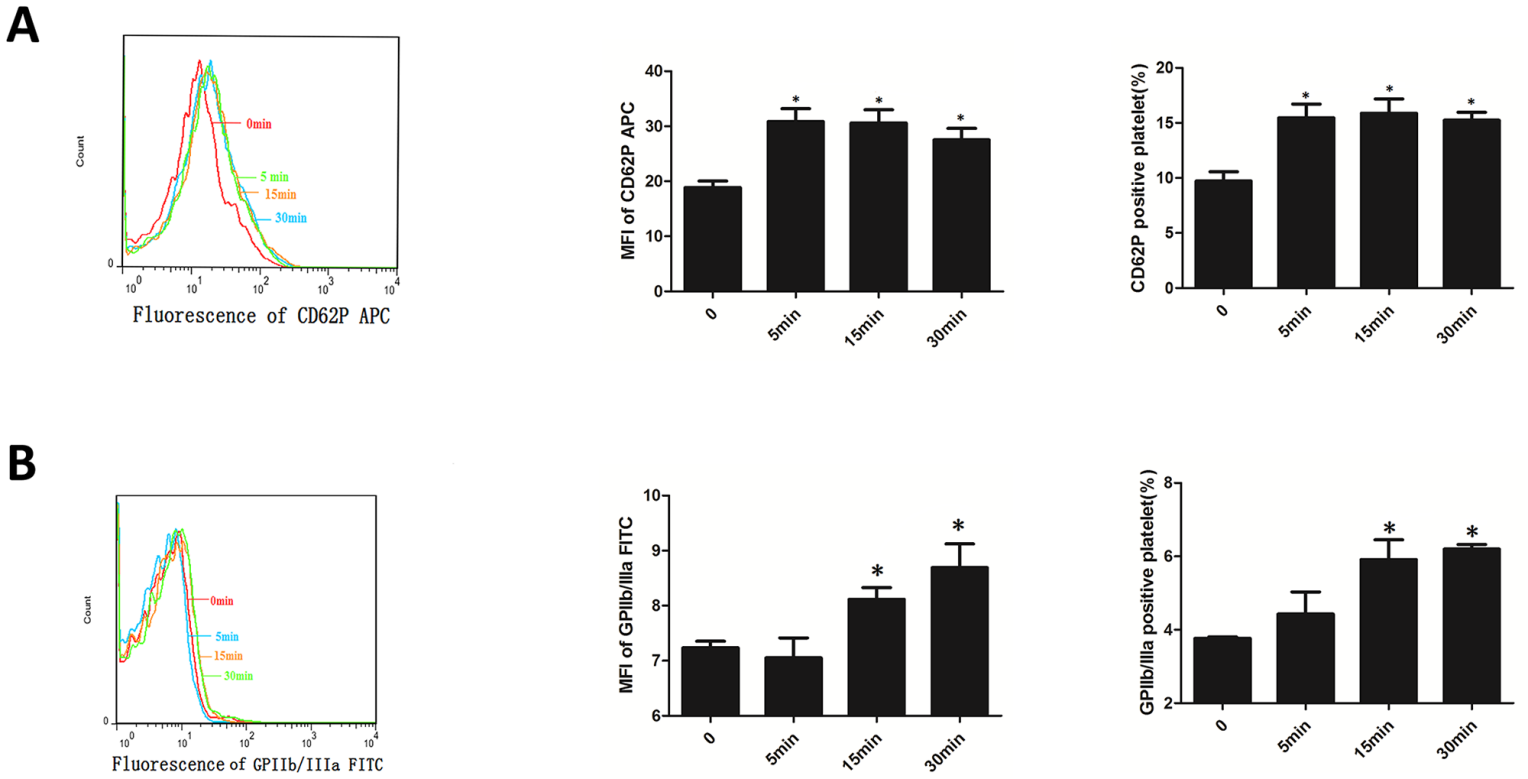
EMPs can activate platelet **A.** Expression of CD62P of platelets which was incubated with EMPs detected by flow cytometry. **B.** Expression of GPIIb/IIIa of platelets which was incubated with EMPs detected by flow cytometry. Histograms represent MFI and positive. *P < 0.05 compared with 0min. Data are means ± SD from at least three separate experiments.

EMPs stimulated platelet, MFI and percentage of platelet CD62P increased from 5min (*P*< 0.05) and maintained a high level at 15min and 30min without difference with 5min (Figure 4A). MFI and percentage of platelet GPIIb/IIIa increased from 15min (*P*< 0.05), and maintained a high level at 30min without difference with 15min (Figure 4B). This suggested that EMPs can activate platelet.

### Platelet stimulated by EMPs expressed PDI and released PMPs

**Figure 5:**
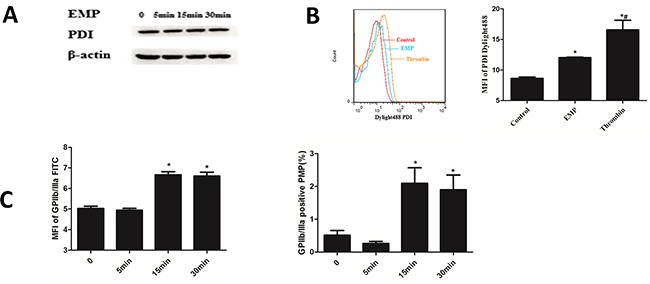
Platelet stimulated by EMPs expressed PDI and released PMPs **A.** Platelets were stimulated with EMPs to detect the platelet PDI expression by Western blotting analysis. **B.** Stimulate platelet with EMPs and 0.5U thrombin to detect the platelet surface PDI by flow cytometry. Histograms represent MFI of PDI Dylight 488. *P < 0.05 compared with control, ^#^*P* < 0.05 compared with EMPs treatment. **C.** Histograms represent MFI and percentage of GPIIb/IIIa in PMPs. *P < 0.05 compared with 0 min. Data are means ± SD from at least three separate experiments.

Platelets were stimulated with EMPs, results showed that platelet PDI expression didn't change with the stimulating time of EMPs longed (*P*>0.05) (Figure 5A). Stimulate platelet with EMPs and 0.5U thrombin, the results showed that platelet stimulated by EMPs and thrombin expressed more PDI than the control group (*P*< 0.05) (Figure 5B). MFI and percentage of GPIIb/IIIa in PMPs increased at 15min (*P*< 0.05) and maintained a high level at 30 min (Figure 5C).

### PDI-dependent EMPs induced platelet aggregation

**Figure 6:**
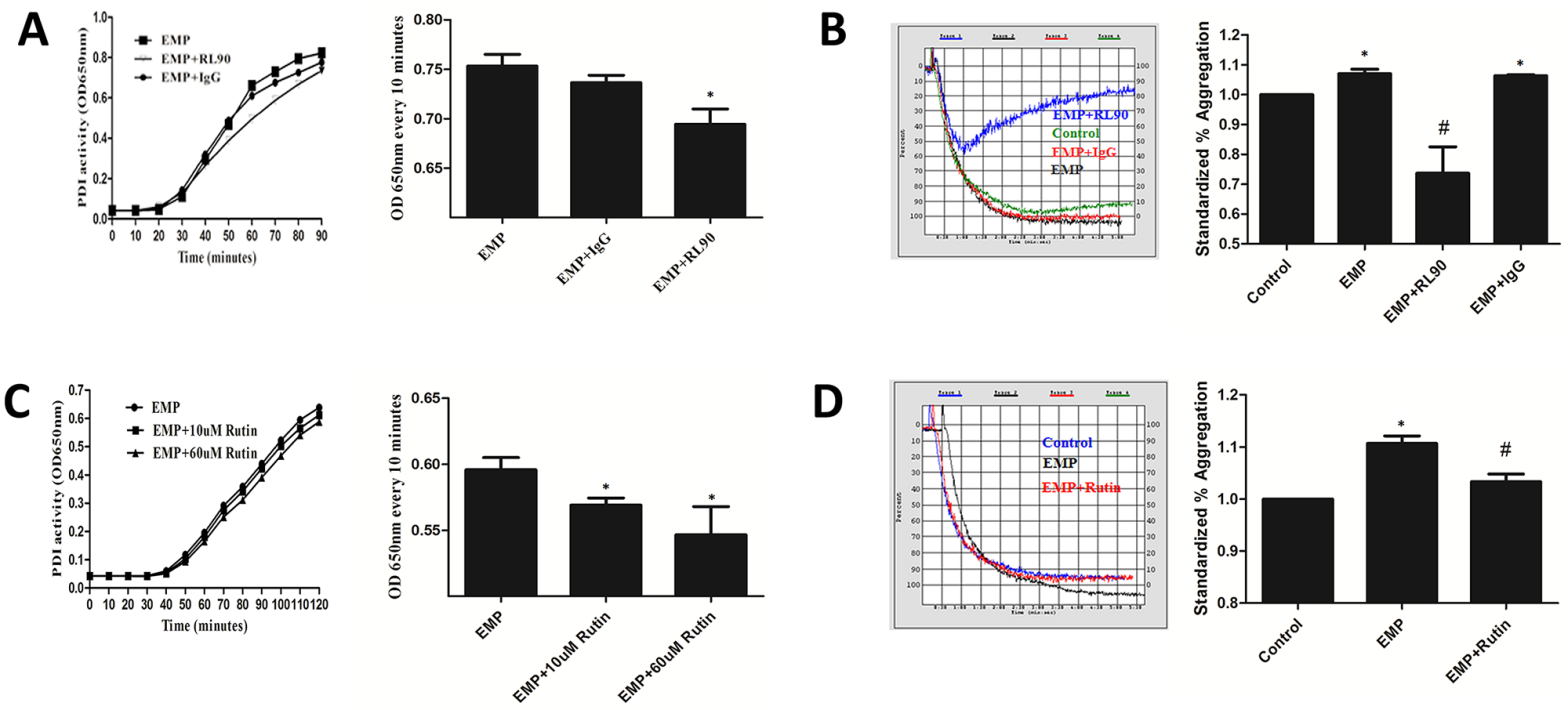
PDI-dependent EMPs induced platelet aggregation Incubate EMPs with 1μg/ml RL90, IgG, 10μM and 60μM rutin at 37°C for 30min. **A, C.** Detect PDI activity of EMPs by insulin transhydrogenase assay, *P < 0.05 compared with EMPs treatment. **B, D.** Detect platelet aggregation by an automatic platelet aggregation meter, *P < 0.05 compared with control, ^#^*P* < 0.05 compared with EMPs treatment. Data are means ± SD from at least three separate experiments.

PDI enzymatic reaction curve of EMPs incubated with RL90 had gentler slope, rised slower, and changes of OD650nm every 10 minutes were lower than EMPs group (*P*< 0.05), which suggested RL90 inhibited PDI activity of EMPs (Figure 6A). Isotype (EMP incubated with IgG) had the same PDI activity with EMPs group (*P*>0.05) (Figure 6A). Stimulate platelet with these EMPs and detect platelet aggregation. Results showed that EMPs group and isotype group had higher maximum aggregation compared with control (*P*< 0.05), EMPs incubated with RL90 had a significantly lower maximum aggregation compared with EMPs group (*P*< 0.05) (Figure 6B). These results suggested that EMPs can promote platelet aggregation and which can be inhibited with RL90.

PDI enzymatic reaction curve of EMPs incubated with rutin (10μM and 60μM) had gentler slope, rised slower, and changes of OD650nm every 10 minutes were lower than EMPs group (*P*< 0.05) (Figure 6C), which suggested different concentration of rutin inhibited PDI activity of EMPs. Stimulate platelet with EMPs incubated with 60μM rutin at 37°C for 30min and detect platelet aggregation. Results showed that EMP group had higher maximum aggregation compared with control (*P*< 0.05), EMP were incubated with 60μM rutin had a significantly lower maximum aggregation compared with EMP group (*P*< 0.05) (Figure 6D).

### PDI was involved in EMP-induced platelet GPIIb/IIIa activity

**Figure 7:**
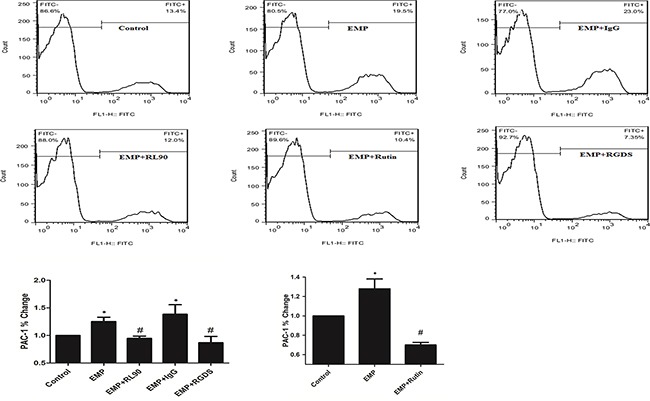
PDI was involved in EMP-induced platelet GPIIb/IIIa activity Incubate EMPs with 1μg/ml RL90, IgG (1μg/mL), rutin(10μM), rutin(60μM) at 37°C for 30min respectively, then stimulate platelet with the EMPs. Or incubate EMPs with RGDS (10μg/mL) with platelet at 37°C for 15min, then incubate the platelet with EMPs. Detect platelet expression of PAC-1 (represented activated GPIIb/IIIa) by flow cytometry. Histograms represent the PAC-1% change. *P < 0.05 compared with control, ^#^*P* < 0.05 compared with EMPs treatment. Data are means ± SD from at least three separate experiments.

Compared with control, EMP group had more expression of PAC-1 (*P*< 0.05) and isotype group (1μg/ml) had more expression of PAC-1 (*P*< 0.05). Compared with EMP group, isotype group had same expression of PAC-1 (*P*>0.05), EMPs were incubated with RL90 group (1μg/ml) or rutin group (60μM) had lower expression of PAC-1 (*P*<0.05), platelet pretreated with RGDS group (10μg/mL) had lower expression of PAC-1 (*P*< 0.05) (Figure 7). These results suggested that PDI was involved in EMP-induced platelet GPIIb/IIIa activity.

## DISCUSSION

Our work demonstrated that the levels of EMPs and PMPs in the plasma of metabolic syndrome patients were significantly increased. At the same time, PDI activity in plasma and on microparticles were increased. More EMPs were secreted from IR HUVECs, and their corresponding PDI enzyme activity increased. EMPs activated platelets and amplified platelet activation by increasing activity of psPDI in a signaling cascade. EMP-PDI could stimulate platelet activation through activating GPIIb/IIIa and this process could be partly blocked by RL90 and rutin.

Previous studies have confirmed that platelet hyper-reactivity/activation exists in MetS [18, 19]. And platelet hyper-reactivity/activation plays a central role in accelerating atherothrombosis and that is the result of the interaction among the cluster of risk factors in MetS: insulin resistance, inflammation, oxidative stress [19, 20]. A large number of studies have confirmed that endothelial dysfunction is increased and more EMPs are released in both the MetS patients and DM patients [21–24], which were consistent with our results. In addition to that, we also found that the level of PMPs increases in MetS. There are a lot of coagulation factors in the surface of particles, which can promote the platelet activation. We found that PDI activity is higher in MetS patients, which suggesting that the process of EMPs and PMPs activating platelets was correlated with PDI. Therefore EMPs may bridge the gap between endothelial dysfunction and platelet activation.

The endothelial cells will release EMPs when they are stimulated or apoptosis [23, 25, 26]. Nevertheless, it is rarely reported the field of the relationship between EMPs and platelet activation. Our study revealed that the IR HUVECs released a large number of PDI-containing EMPs, and EMP-PDI activity was significantly enhanced. When platelets were incubated with these EMPs, we found that the expression level of CD62P, PAC-1 (platelet activation markers) in these platelets were significantly increased, which suggesting that IR-EMPs can significantly activate platelets. Arun Raturi et al [17] have shown that platelet microparticle-associated PDI promoted platelet aggregation, and transfered the information of IR in T2DM. Thus, EMPs may also activate platelet through PDI and transfer the information of IR.

Integrin β3 mediates platelet adhesion and aggregation, and the activation of integrin β3 is the final common pathway of platelet activation. The isomerism of integrin β3 is the key step of platelet GPIIb/IIIa activation. On the surface of platelets, GPIIb/IIIa is expressed as α_IIb_β_3_[27], and on the surface of endothelial cells it is α_V_β_3_ not α_IIb_β_3_[28]. PDI plays an important role in the process of platelet GPIIb/IIIa activation [29–31]. A study about the β3-null (β3^−/−^) mice has shown that PDI capture during thrombus formation in vivo depends on the presence of β3 integrin [30]. This is also supported by our observation that platelets GPIIb/IIIa activation were significantly reduced when platelets were incubated with the PDI inhibitors RL90. Therefore, we expect that PDI plays a crucial role in the activation of platelet GPIIb/IIIa and platelet aggregation. Jasuja and Furie [31] also have proved that it is the endothelium-derived but not platelet-derived PDI that is required for thrombus formation in vivo. This is consistent with our results.

Several studies have confirmed that psPDI is important for platelet release and platelet aggregation. Jasuja and Furie [31] have shown that platelet PDI contributes to the total amount of thrombus-associated PDI. And Kim Hahm et al [32] have confirmed that platelet PDI is important for thrombus formation. Our work also confirmed that when platelets were activated by EMPs, the expression of psPDI increased and platelets released more PMPs to achieve signal cascade.

## CONCLUSION

GPIIb/IIIa receptor on the surface of platelets can be activated by EMP-PDI which is released by IR HUVECs, and PDI can be blocked by RL90. And platelet can express more psPDI and release PMPs to achieve signal cascade in MetS.

## MATERIALS AND METHODS

### Materials

The antibodies rabbit anti-RS1, anti-Akt, anti-Ero1α and mouse anti-PI3K and anti-p-Akt were purchased from Cell Signaling Technology (Danvers, MA, USA). The antibodies mouse anti-Integrin β3, Anti-PDI antibody (RL90), IgG isotype control and DyLight ®488–PDI were from Abcam (Boston, MA, USA). The antibody mouse anti-β-actin was purchased from Zhongshanjinqiao(Beijing, China), fluorescein isothiocyanate (FITC)-conjugated anti-PAC-1, phycoerythrin (PE) -conjugated anti-CD31, PE-conjugated isotype control, FITC-conjugated isotype control, PE-Cy5-conjugated anti-CD41a, and allophycocyanin (APC) -conjugated anti-CD62P were purchased from BD Biosciences Pharmingen (San Diego, CA, USA). Prostaglandin E_1_ (PG-E_1_), thrombin and RGDS (PDI inhibitor) were purchased from Sigma (Aldrich, St.Louis, MO, USA). Phosphate Buffered Saline (PBS), Bovine Serum Albumin (BSA), Dithiothreitol (DTT), and bovine insulin were purchased from Solarbio company (Beijing, China), rutin (PDI inhibitor) was purchased from Tokyo Chemical Industry (Tokyo, Japan), DMEM was purchased from Gibco Company (Carlsbad, CA, USA). Fetal Bovine Serum (FBS) was purchased from Hyclone (Logan, UT, USA). The monoclonal antibody AD-1 for microparticles was offered by Jingti Deng professor in Department of Biochemistry and Molecular Biology of University of Calgary in Canada. And analyzed on FACSCalibur and FlowJo software (BD Biosciences, San Jose, CA), microplate reader (Thermo Scientific, Waltham, MA, USA), dual channel aggregometer (Chrono-log Corp., Havertown, PA, USA), and Z2 particle counter (Beckman Coulter, Fullerton, CA, USA), transmission electron microscope (TEM) JEOL-1200EX(JEOL, Japan).

### Selection of clinical cases and sample collection

Twenty-three patients with MetS and eight normal healthy controls were enrolled after appropriate inform consent was obtained. Select clinical cases according to a joint interim statement of MetS diagnosis by IDF/AHA/free of any medication for 2 wks. Written consent was obtained from all donors, and protocols were approved by the institutional ethics committee. The subjects were fasting overnight for 8~10 hours.

### Platelet preparation

Platelet preparation is described previously [33]. Briefly, draw blood from median cubital vein of all donors to anticoagulation tubes with 0.109 mol/L sodium citrate. Platelet-rich plasma (PRP) was prepared by centrifugation at 120g for 10min at room temperature. Platelets were isolated from PRP after centrifugation at 800g for 10min in the presence of acid citrate dextrose. Then platelets were washed and resuspended in modified Tyrode's buffer (137 mmol/L NaCl, 2.7 mmol/L KCl, 12 mmol/L NaHCO_3_, 0.4 mmol/L NaH_2_PO_4_, 5 mmol/LHEPES, 0.1% glucose and 0.35% bovine serum albumin, pH7.2) in the presence of 100 nmol/L PG-E_1_. Platelet concentration was adjusted to 1 × 10^6^/mL by use of a Z2 particle counter, then used at once in experiments.

### Human umbilical vein endothelial cell culture and build IR model

HUVECs were presented by Gynecologic Oncology Laboratory of Qilu Hospital. Culture HUVECs by low glucose DMEM complete medium (containing 10% FBS, 400 μl/ml growth factor and antibiotics). Select conditions by culture HUVECs under conditions of low glucose, low glucose combined with high insulin, high glucose, high glucose combined with high insulin. Select timing by culture HUVECs under high glucose combined with high insulin for 0, 2h, 6h, 12h, 24h.

### Identification of EMPs

#### EMPs Isolation

EMPs were isolated from the supernatant of HUVECs after centrifugation at 15000g for 1hour at the 4°C, in total the EMPs were resuspended in the PBS and stored at −80°C.

#### Assessment of EMPs by flow cytometry and scanning electron microscopy

The HUVEC suspension (2.5μL) was incubated in the dark at room temperature for 15 min with 2ul PE-CD31 or IgG isotype controls for PE, then they were washed with PBS. For each sample, a total of 20000 events in the EMPs gate were collected and analyzed by using a FACSCalibur flow cytometer. The structure of EMP is observed by TEM.

#### Assessment of EMP-PDI by flow cytometry

The HUVEC suspension (2.5μL) was incubated in the dark at room temperature for 15 min with 2 μL PE conjugated anti-CD31 antibody and 2μL Dylight488 conjugated PDI antibody, then they were washed with PBS. For each sample, a total of 20000 events in the EMPs gate were collected and analyzed by using a FACSCalibur flow cytometer.

#### ELISA detection of total amount of EMPs

ELISA detection of total amount of EMPs was performed as described previously [33]. Briefly, the goat anti Mouse IgG was diluted to 10μg/mL by package buffer(10mM Tris, 10mM NaCl), every well was added 50μL goat anti Mouse IgG for one night at room temperature. After specific capture for microparticles, the plate coated with AD-1 was added to well with 50 μL/well at 4°C for one night, then after 1 washing with 0.05% PBST, 50 μL of supernatant of HUVECs was added to each well and incubated for 2h at 37°C, after 6 washings, the linear absorbance was recorded at 450 nm by use of a microplate reader after the addition of substrate solution. Then, let them incubate for 1h at 37°C, the plate was read at 450 nm. Quantification of EMPs is obtained by the difference value of the two value of 450 nm.

### Platelet functional studies

#### Flow cytometry detection of platelet activation

We detected the platelet activation after the platelet was incubated with EMPs (30μg/ml) by flow cytometry. Incubate EMPs with 1μg/ml RL90, IgG (1μg/mL), rutin(10μM), rutin(60μM) at 37°C for 30min respectively, then stimulate platelet with the EMPs. Or incubate EMPs with RGDS (10μg/mL) with platelet at 37°C for 15min, then incubate the platelet with EMPs. The suspension (20μL) was incubated with 2μL APC-conjugated anti-CD62P antibody and 2μL FITC-conjugated PAC-1 antibody in the dark at room temperature for 15 min then they were washed with PBS. For each sample, a total of 20000 events in the platelet gate were acquired and analyzed by using a FACSCalibur flow cytometry.

#### Platelet aggregation

Aggregation was performed as described previously [33]. Briefly, aggregation was assessed by turbidimetry with a dual channel aggregometer, with 2 μmol/L ADP used as an agonist as. PRP was obtained by centrifuging whole blood at 120g for 10 min at 22°C, and platelet-poor plasma (PPP) was obtained by centrifuging PRP at 3000g for 10 min. The platelet concentration of PRP was adjusted to 2.5 × 10^8^/mL by the addition of PPP. An amount of 100% aggregation was defined as the light transmission of PPP, and 0% was defined as the light transmission of PRP before the addition of agonists. Then the PRP was stimulated with 2 μmol/L ADP, and the change in light transmission was recorded.

#### The PDI activity by insulin transhydrogenase assay

PDI activity was determined using the insulin transhydrogenase assay [34]. Add 250μL (1mg/mL) insulin and 50μL human plasma or the cell supernatant incubated with RL90, IgG, 10μM and 60μM rutin, then add 10μL DTT (100mM) to a 96-wells plate. Mix gently. Detect absorbance at 650nm at once and every 10 minutes by a microplate reader for continuous 90~120 minutes.

#### The EMP-PDI activity by insulin transhydrogenase assay

Firstly, the monoclonal antibody AD-1 was bound to a 96-well plate, then add 50μL the cell supernatant for 2hours in 37°C. After 6 washing, add 100μL (1mg/mL) insulin and 3μL DTT (100mM), to this plate and mix gently. Detect absorbance at 650nm at once and every 10 minutes by a microplate reader for continuous 90~120 minutes.

#### Western blot analysis

HUVEC lysates was subjected to immunoblotting with antibodies against IRS-1 or PI3K or p-Akt or total Akt or β-actin and platelet lysates was subjected to immunoblotting with antibodies against PDI or β-actin, followed by anti-IgG horseradish peroxidase–conjugated secondary antibody.

### Statistical analysis

Data are expressed as mean ± SD and analyses involved use of GraphPad Prism 5 (La Jolla, CA, USA). Differences among groups were analyzed by one-way

ANOVA followed by Tukey post-hoc test. *P* < 0.05 was considered statistically significant. Analyses involved use of SPSS 18.0 (SPSS Inc. Chicago, IL, USA).

## SUPPLEMENTARY MATERIAL


