# Supplementary material for: Endothelial cells microparticle-associated protein disulfide isomerase promotes platelet activation in metabolic syndrome
Source: Oncotarget. 2016 Nov 4;7(50):83231–40. doi: 10.18632/oncotarget.13081 (PMC5347765; doi:10.18632/oncotarget.13081)
Supplement: Supplementary file 1 [file oncotarget-07-83231-s001.pdf]

Endothelial cells microparticle-associated protein disulfide isomerase promotes platelet activation in metabolic syndrome

SUPPLEMENTARY MATERIAL

Representative images of HUVECs under all experimental conditions are shown in supplement material. HUVECs displayed a cobblestone-like shape. There were no morphological differences between all condition HUVECs. Representative images of HUVECs under all experimental conditions.

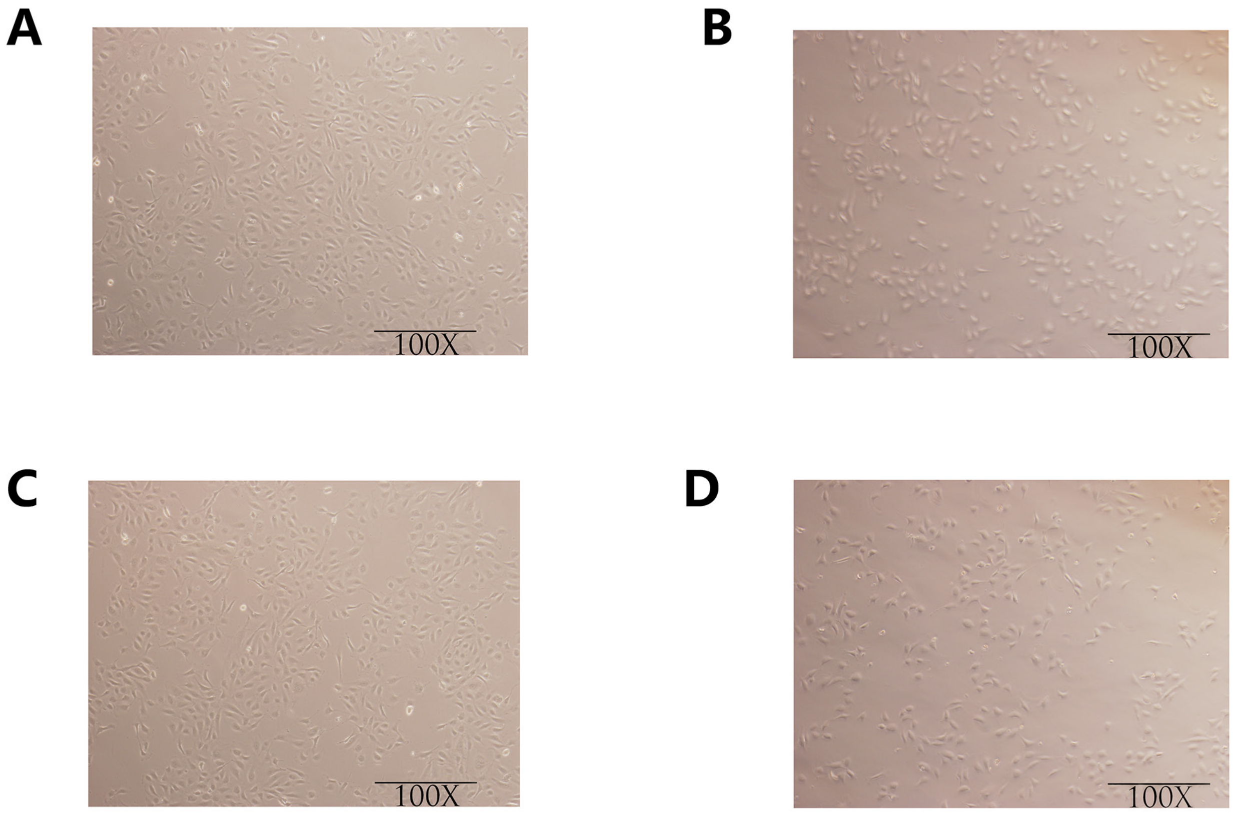

Supplementary Figure S1: Representative images of HUVECs under all experimental conditions. Culture HUVECs by low glucose DMEM complete medium under conditions of low glucose(LG) **A.** low glucose combined with high insulin(LG+INS) **B.** high glucose(HG) **C.** high glucose combined with high insulin (HG+INS) **D.** for 24 hours.
